# Transactions of the American Medical Association

**Published:** 1850-11

**Authors:** 


*Transactions of the American Medical Association.* Vol. III.—The volume of Transactions of the American Medical Association for 1850 has been received, too late for notice in this No. beyond the announcement of its publication. Copies may be obtained on application to Messrs. Lea & Blanchard, Philadelphia. Societies which have been represented in the Association will be furnished copies for the members on the following terms:—3 copies of Vol. III, in paper covers, for $5.; 3 copies of Vols. I, II, and III, for $12.

